# Single institutional experience of peripheral applications of a liquid embolic agent: Ethylene Vinyl Alcohol Copolymer

**DOI:** 10.1186/s42155-020-00117-2

**Published:** 2020-08-02

**Authors:** Abhijit L. Salaskar, Faezeh Razjouyan, Alexander L. Cho, Rishi R. Sood, Andrew Akman, Daniel Scher, Anthony C. Venbrux, Shawn N. Sarin

**Affiliations:** Division of Vascular and Interventional Radiology, George Washington University Hospital, Washington, DC 20037 USA

**Keywords:** Ethylene vinyl alcohol copolymer, EVOH, Onyx embolization, Arteriovenous malformations, Endoleak type II, Pseudoaneurysm

## Abstract

**Objective:**

To evaluate the safety and efficacy of ethylene vinyl alcohol (EVOH) copolymer for the treatment of a variety of peripheral vascular pathologies.

**Results:**

Between October 2010 and October 2017, 43 patients who underwent total 54 EVOH embolization procedures for the treatment of peripheral vascular pathologies were included. The cases which involved the use of EVOH for the treatment of nonvascular, neurologic, ophthalmologic, otolaryngologic or head-neck pathologies were excluded. The demographic data, technical and clinical success rates, and procedure-related details and complications were obtained. The most common indications for EVOH embolization were type II endoleaks (*n* = 18) and peripheral arteriovenous malformations (*n* = 14). The majority of cases (62.5%) used EVOH without any adjunct embolic material. The results of this study showed 100% technical success rates and 89% clinical success rates. No events of nontarget embolization or other procedure-related complications were noted. The mortality & morbidity rates were 0%. The loss to follow up rate was 16% (9 /54). The mean follow-up period was 134 days (range, 30 to 522 days).

**Conclusion:**

The single institutional experience supports the safety and efficacy of EVOH embolization in the treatment of various peripheral vascular conditions.

## Background

Ethylene Vinyl Alcohol Copolymer, EVOH (Onyx™, Medtronic, Minneapolis, MN) is a non-adhesive, non-absorbable, permanent liquid embolic agent. The use of EVOH for preoperative embolization of cerebral arteriovenous malformations (AVMs) has been FDA approved since 2005 (Loh & Duckwiler, 2010), and has since expanded to treat AVMs and aneurysms in the head, neck, and spine. However, the use of EVOH for the treatment of peripheral pathologies remains limited. In the periphery, it has been used in the treatment of type II endoleaks (EL-2) (Massis et al., 2012) and peripheral AVMs (Cantasdemir et al., 2012; Numan et al., 2004). Other rare off-label uses of EVOH include embolization of gastrointestinal hemorrhage (Urbano et al., 2014), post-traumatic hemorrhage (Müller-Wille et al., 2011), type I endoleaks (Eberhardt et al., 2014), pulmonary hemorrhages (Bommart et al., 2011; Izaaryene et al., 2016), pseudoaneurysms (PSAs) (Vanninen & Manninen, 2007), pelvic AVMs (Castaneda et al., 2002; Wohlgemuth et al., 2015), and neoplasms. (Regine et al., 2015) The existing literature is in the form of case reports or series. The possible reasons for the slow adoptation of this agent in the treatment of peripheral pathologies could be secondary to its perceived technical complexity, inadequate training, feared complications, high cost and lack of data demonstrating its efficacy and safety profile. The purpose of this retrospective review was to evaluate the safety and efficacy profile of EVOH in the treatment of peripheral vascular conditions and contribute the results of this retrospective review to an existing literature.

## Methods

Institutional database was retrospectively reviewed to identify all patients who were treated with the use of EVOH between October 2010 and October 2017. We excluded cases involving nonvascular, neurologic, ophthalmologic, otolaryngologic or head-neck pathologies. The study was approved by the Institutional Review Board. All the patients had provided informed consent. From our database, we obtained demographic data, technical and clinical success rates, and procedure-related details, including embolic agents used, complications (Cx), and clinical outcomes.

### Embolization technique

Standard EVOH embolization techniques were strictly employed as previously described in detail (Guimaraes & Wooster, 2011; Kilani, 2015). The desired targets of embolization were approached with DMSO compatible microcatheters.

### Study endpoints

Technical success (TS) was defined as the lack of active contrast extravasation beyond the site of embolization, and as elimination of flow to the aneurysmal sac or absence of residual endoleak in cases of endoleak embolization along with an absence of non-target embolization. Clinical success (CS) in cases of endoleak embolization was defined as decrease or stabilization of aneurysm size on a post embolization CT. Clinical success in cases other than endoleak embolization was defined as the resolution of signs and symptoms within 30 days of embolization and without major procedure-related complications. Embolizations requiring multiple sessions such as venous malformations (VM) or endoleaks were considered separate cases for a given patient, and not classified as clinical failures. Determination of success rates and complications are consistent with definitions put forth by the Society of Interventional Radiology Committee and CIRSE. (Angle et al., 2010; CIRSE, 2017)

## Results

Over a period of 7 years, we identified 43 patients who underwent embolization with EVOH for peripheral vascular pathologies. These 43 patients underwent a total of 54 embolization procedures that met the inclusion criteria. The incongruent number of cases compared to number of patients was due to multiple embolization sessions for several patients. Such cases included: VMs (*n* = 2), AVMs (*n* = 9 among 3 patients), and EL-2 (*n* = 10 procedures for 5 patients). The case etiologies are summarized in Table 1. The choice of embolic agents and viscosity of EVOH was up to the board-certified interventional radiologist’s discretion. The indication for embolization, amount and concentration of EVOH, prior embolization, and prior embolic agents are listed in Table 2. The majority of cases (62.5%) used EVOH without any adjunct embolic material. The adjunct embolic agent used are shown in Table 3. The embolic agents are categorized by etiology in Table 4.

**Table 1:** Patient demographic and case etiologies (*n* = 54)

Patients (*n* = 43)	
Male	33 (76%)
Female	10 (24%)
Mean age	57.07 (± 22 years Standard Deviation)
Cases (*n* = 54)	Frequency
Type II endoleak	18
Peripheral AVM	14
Pseudoaneurysm	11
Peripheral VM	4
Peripheral AV fistula	2
Yttrium-90 angiography mapping	2
Malignancy	1
Peripheral aneurysm	1
Acute gastrointestinal hemorrhage	1

**Table 2:** Features of cases treated with EVOH (*n* = 54)

Indications for treatment (%)	
Primary treatment	44 (81.4%)
Pre-surgical devascularization	5 (9.2%)
Palliative or symptomatic relief	5 (9.2%)
Mean volume of EVOH	2.2 ml ± 2.3
Type of EVOH concentration used (%)	
18	13 (24%)
34	35 (65%)
Both	6 (11%)
Cases with prior embolization and type of agents used (%)	
No prior treatment	32 (59.2%)
Onyx	9 (16.6%)
Coils	5 (9.2%)
Coils + another agent	5 (9.2%)
+ Onyx	2
+ Thrombin	1
+ Gel foam	1
+ Gel foam & Onyx	1
Gel foam	1 (1.8%)
Onyx + *n*-butyl cyanoacrylate (NBCA)	1 (1.8%)
Microspheres	1 (1.8%)

**Table 3:** Adjunctive embolic agents that were used with EVOH

Agent	Frequency (%)
None (EVOH only)	34 (62.9)
Coils	12 (22.2)
N-butyl cyanoacrylate (NBCA)	4 (7.4)
Sotradecol foam	2 (3.7)
Embospheres	1 (1.8)
Coils & Gel foam	1 (1.8)

**Table 4:** Embolization agent based on case etiology

Etiology (n = number of cases)	N (%) Embolic agents
Type II endoleak (n = 18)	14 (78%) EVOH alone
	4 (22%) EVOH + coils
Peripheral AVM (n = 14)	
Pelvic / Uterine AVMs (n = 7)	6 EVOH alone
	1 EVOH + coils
Extremity AVMs (n = 7)	5 EVOH alone
	2 EVOH + Sotradecol foam
Pseudoaneurysm (n = 11)	5 EVOH + coils
	6 EVOH alone
Peripheral venous malformation (n = 4)	4 EVOH + NBCA
Peripheral arteriovenous fistula (n = 2)	1 EVOH alone
	1 EVOH + Coils & Gelfoam
Yttrium-90 angiography mapping (n = 2)	2 EVOH alone
Malignancy (n = 1)	1 EVOH + Embosperes
Peripheral aneurysm (n = 1)	1 EVOH alone
Acute gastrointestinal hemorrhage (n = 1)	1 EVOH alone

**Fig. 1:**
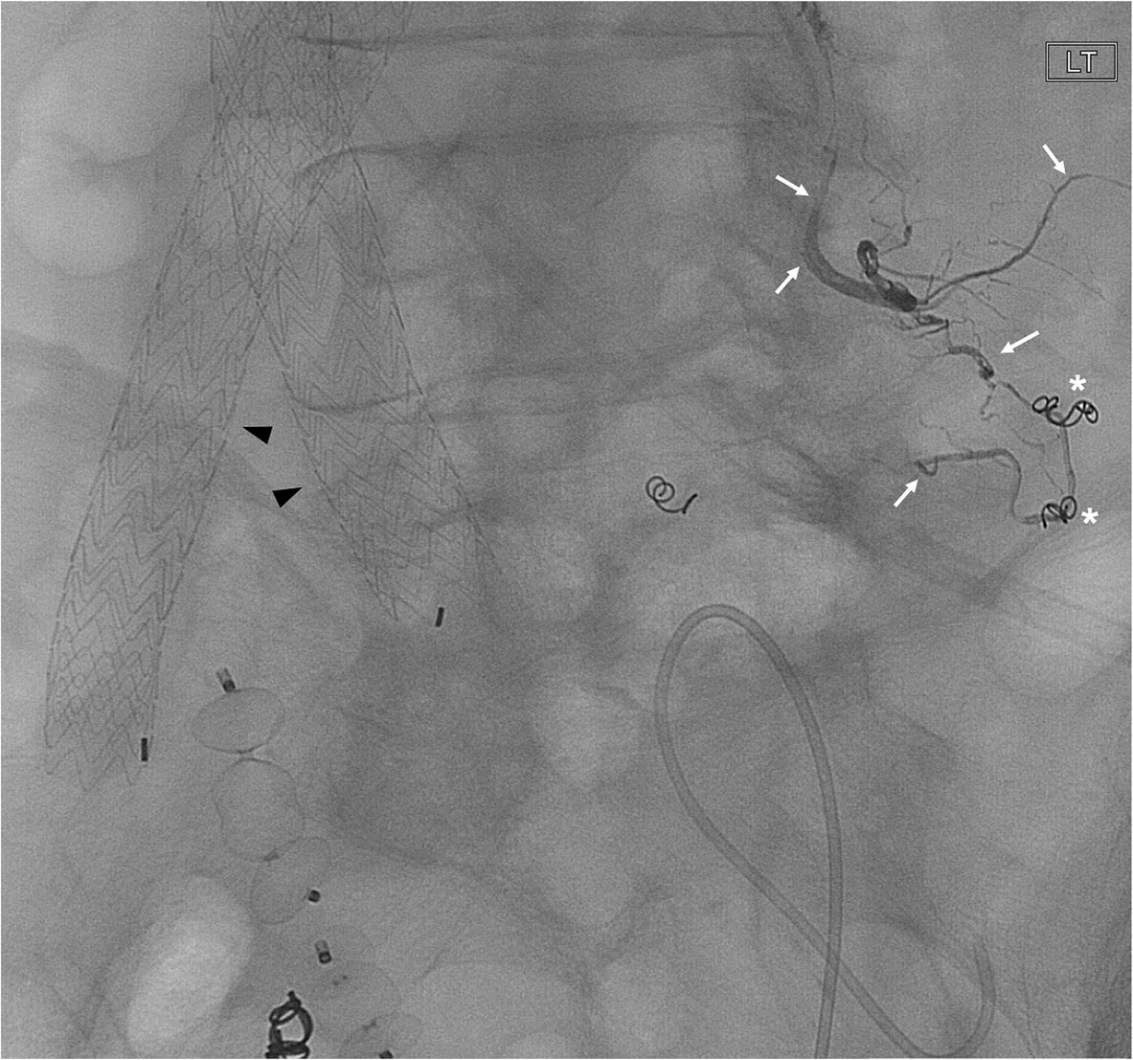
75 year-old male with an enlarging AAA sac status post endovascular repair (arrowhead). Fluoroscopic image demonstrates EVOH (Onyx 34) embolization of a third order branch of L4 Lumbar artery (arrows) which was leading to type II endoleak. Prior attempt of coil embolization of this endoleak was unsuccessful (asterisk)

**Fig. 2:**
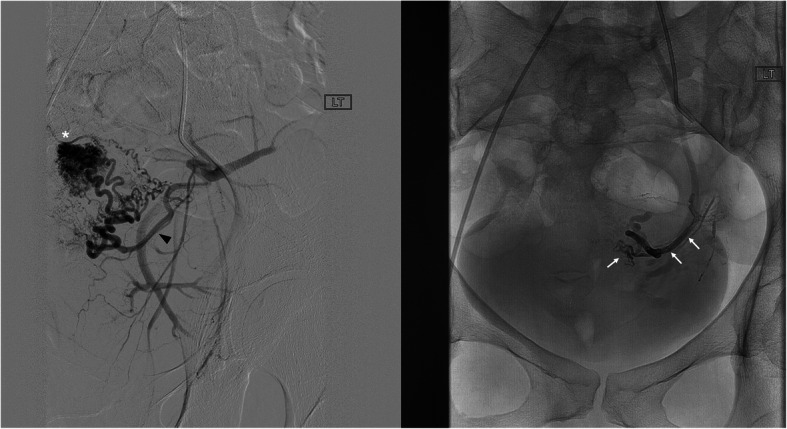
**a** 40 year old female who presented with heavy vaginal bleeding. Fluoroscopic image demonstrates a high flow, large uterine AVM (asterisk) supplied by a left uterine artery (arrowhead) and drained by veins. **b**. Fluoroscopic image shows successful EVOH embolization of the left uterine artery and non-opacification of AVM (arrow)

**Fig. 3:**
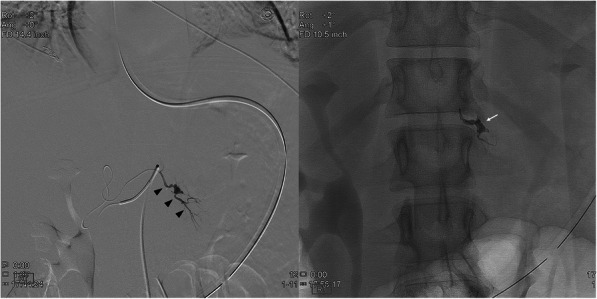
**a** 28 year-old male presenting with RUQ abdominal pain. Selective angiogram of a left hepatic artery branch demonstrates a PSA (arrowhead). **b**. Fluoroscopic image shows EVOH (Onyx 18) embolization leading to occlusion of afferent, efferent vessel and sac of PSA (arrow)

**Fig. 4:**
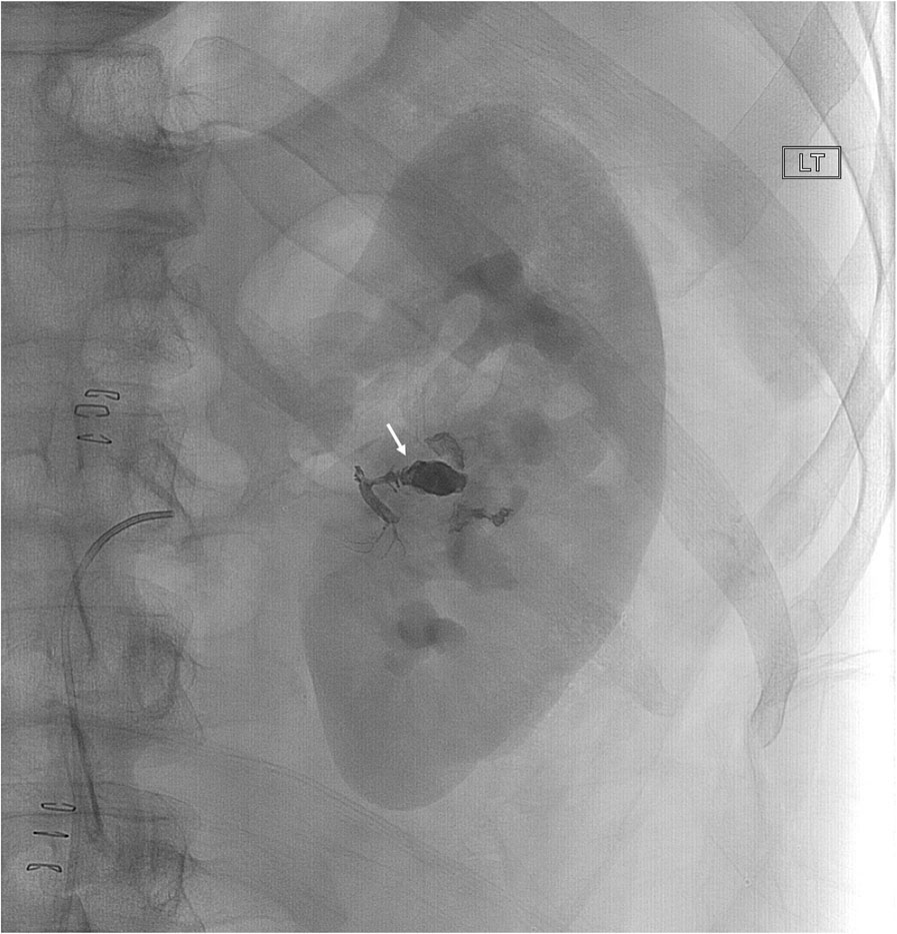
53 year-old male presented with hematuria after left partial nephrectomy. Fluoroscopic image demonstrates successful EVOH (Onyx 34) embolization of afferent, efferent vessel and sac of the PSA (arrow), arising from a third order branch of a left renal artery

**Fig. 5:**
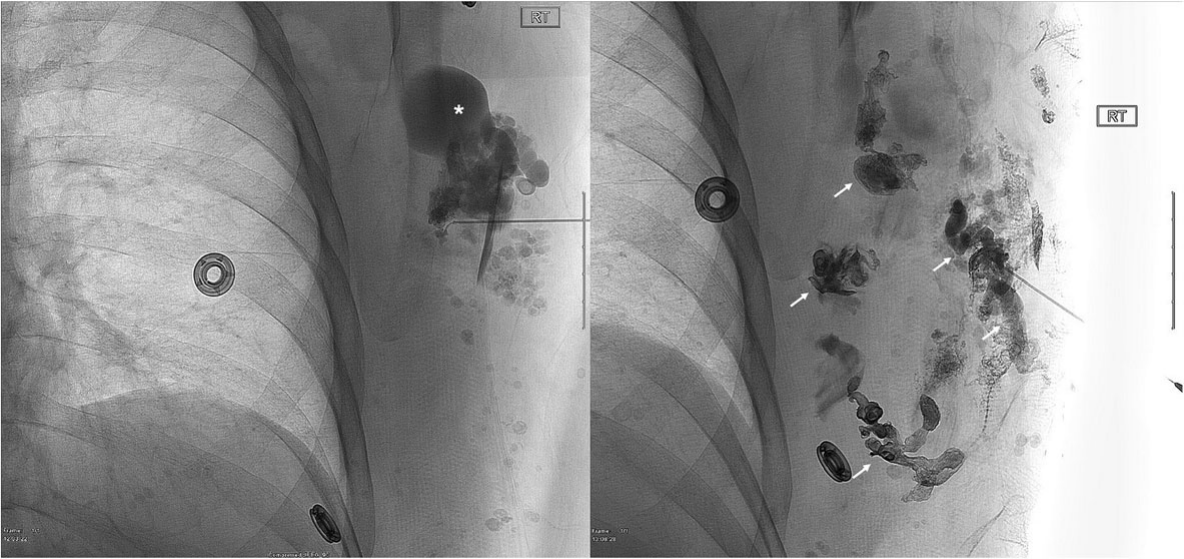
**a** 61 year-old male presented with swelling involving the right shoulder, and pectoral region secondary to VM. Fluoroscopic image demonstrates a large VM (asterisk). **b**. Fluoroscopy image after percutaneous embolization shows filling of venous lakes in the VM by embolic agents (arrows)

**Fig. 6:**
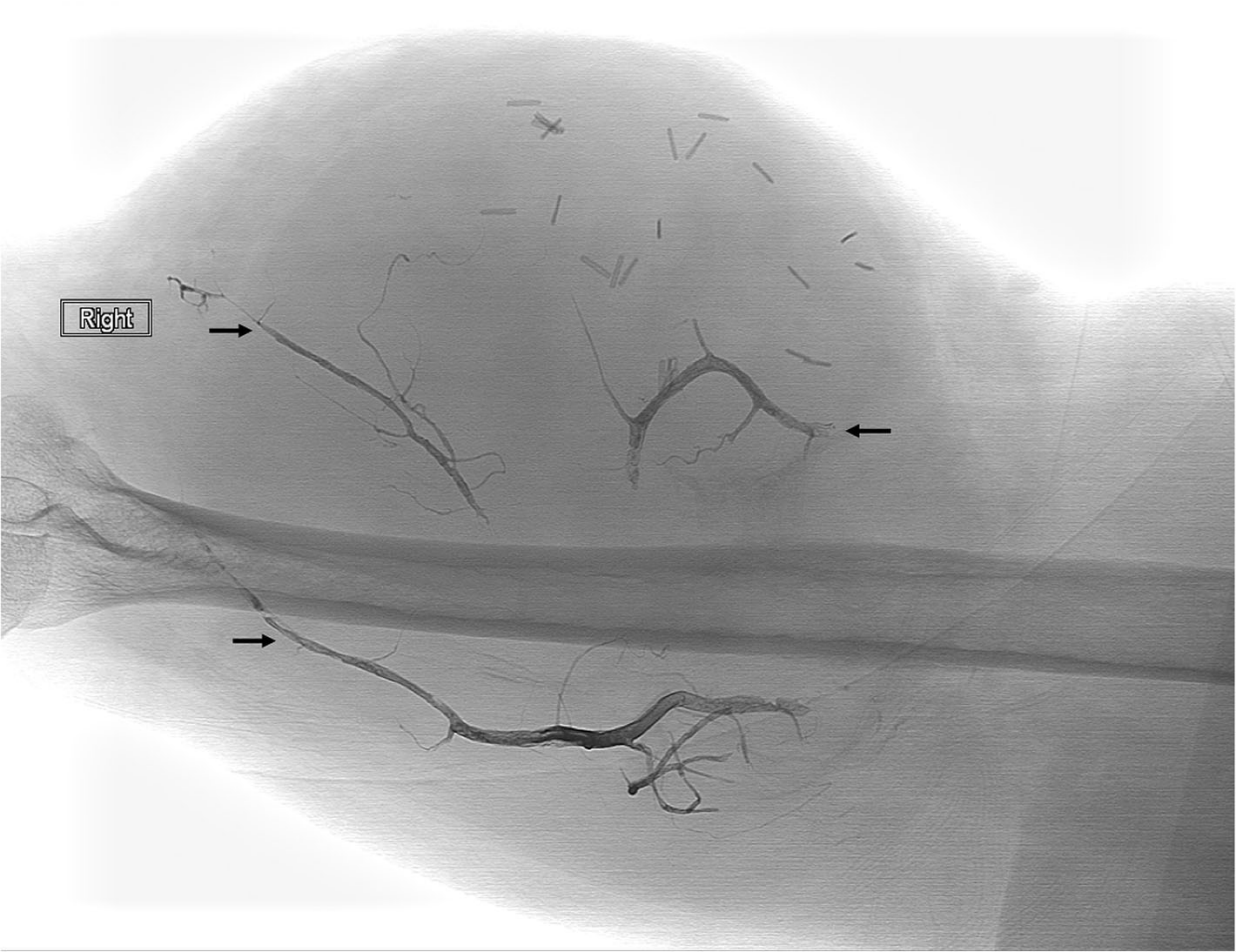
54-year-old male presented for devascularization of right arm sarcoma prior to surgical resection. Fluoroscopic image demonstrates filling of arterial branches in the upper arm mass by EVOH (arrows) and paucity of vascularity following EVOH and embosphere embolization of the brachial artery branches

**Fig. 7:**
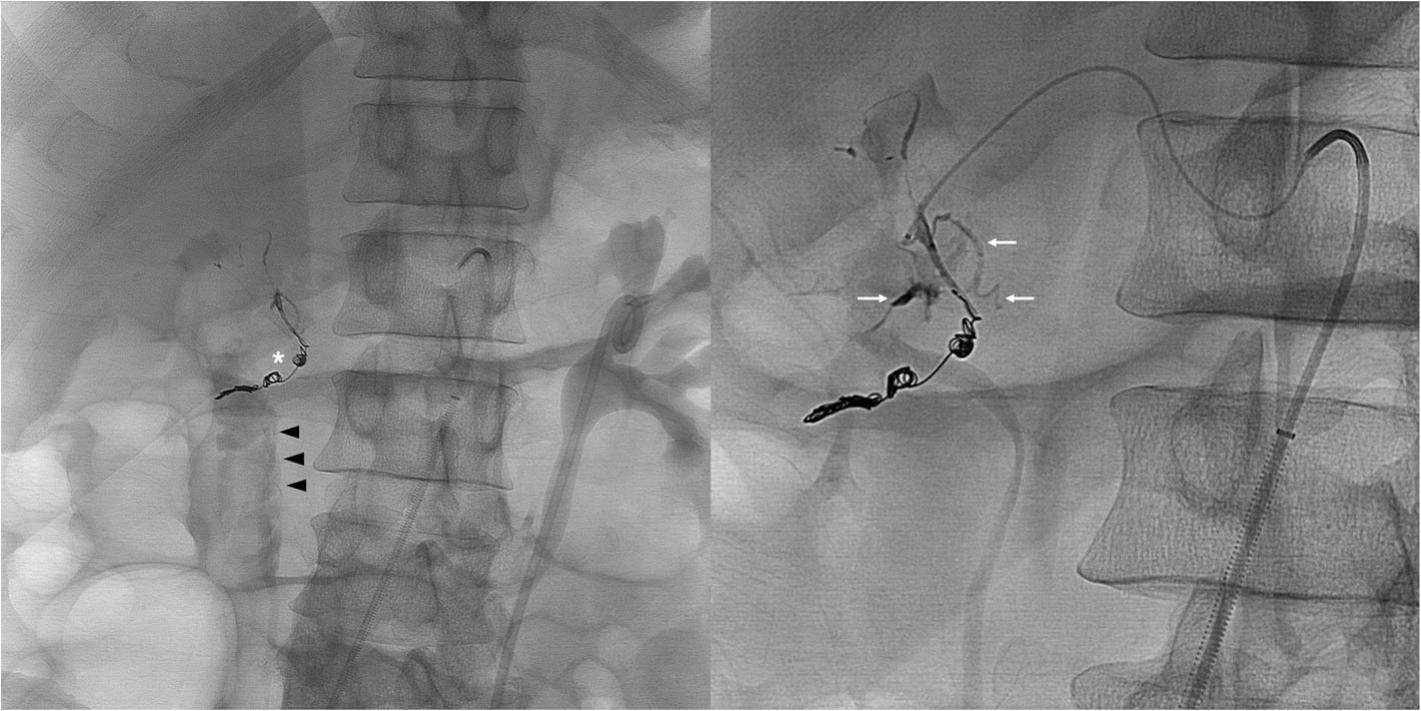
**a** 57 year old male presented with hematemesis and melena. Fluoroscopic image demonstrates persistent active extravasation of the contrast into proximal duodenum (arrowhead) despite deployment of four fibered platinum detachable microcoils (asterisk). **b** The fluoroscopic image demonstrates selective angiography of gastroduodenal artery and absence of extravasation after EVOH embolization (arrow)

The patient characteristics, presenting symptoms/signs, embolization targets, EVOH concentrations, adjunct embolic agents, procedure details, outcomes are shown in Table 5 for the patients with endoleak embolization; Table 6 for the patients with AVM embolization; Table 7 for the patients with PSA embolization; and Table 8 for patients who underwent embolization for the remaining etiologies. Overall post embolization TS rate was 100%. The CS rate was 89%, which excluded patients lost to follow up and patients who underwent surgical resection after embolization. No events of nontarget embolization (NTE) or other complications were noted. The loss to follow (LTF) up rate including those who received surgical resection after embolization was 16% (9/54). The mean follow-up period was 134 days (range, 30 to 522 days). (Figs. 1, 2, 3, 4, 5, 6, 7)

**Table 5:** Etiology: Type 2 endoleaks

No.	Age/Sex	Location of aneurysm	Presenting Symptoms (Sx)/sign	Approach	Artery or target embolized	EVOH Conc. (ml)	Adjunct embolic	Technical Success (TS)	Clinical Success (CS)	Non Target Embolization (NTE)	Complications (Cx)	Follow up (FU) CT (days)
1	75 M	Infr-Renal Abdominal aortic aneurysm (IR-AAA) (Fig. 1)	↑AAA sac	Trans-Arterial (TA)	Left L4 Lumbar artery (A.) branches via iliolumbar A.	34(1)	N	Yes (Y)	Y	No (N)	N	90
2	72 M	IR AAA	↑AAA sac	Trans-Lumbar (TL)	AAA sac at L3, reflux into L2 arteries	34(3)	N	Y	Y	N	N	30
3	68 M	IR AAA	↑AAA sac	TL	AAA sac, Lumbar A.	34(3)	N	Y	Y	N	N	204
4	69 M	IR AAA	↑AAA sac	TA	AAA sac & Lumbar A. (L4)	34(3)	N	Y	Y	N	N	208
5	88F	Right common iliac artery aneurysm (R CIAA)	↑R CIA sac	TA	Right circuflex iliac A. branch	34(1)	N	Y	Y	N	N	375
6	84 M	IR AAA	↑AAA sac (8 cm)	TA	Lumbar A. (L2)	34(0.6)	N	Y	Y	N	N	522
7	86 M	IR AAA	Chest-abd pain, ↑AAA sac	TA	Post A. feeding AAA sac, Left hypogastric A. & AAA sac	34(4) + 18(1)	N	Y	Y	N	N	40
8	87 M	IR AAA	↑AAA sac	TA	AAA sac	34(6)	N	Y	Y	N	N	29
9	88 M	IR AAA	↑AAA sac	TA	Right iliolumbar A.	34(1)	N	Y	Y	N	N	174
10	81 M	IR AAA	↑AAA sac	TA	AAA sac	34(9)	N	Y	Y	N	N	418
11	84 M	IR AAA	↑AAA sac	TA	Right iliolumbar A.	18(0.5)	N	Y	Y	N	N	105
12	77 M	IR AAA	↑AAA sac	TA	AAA sac	34(6)	N	Y	Y	N	N	36
13	76 M	R CIAA	↑R CIA sac	TA	Right superior gluteal A., Inferior division of right IIA, main trunk of right IIA & CIA	34(2)	Coils	Y	Y	N	N	54
14	53 M	Right internal iliac artery (R IIAA)	↑ R IIA sac, abdominal pain	TA	Four collateral branches of inf. Division of R Lumbar A.(L4)	34(0.5)+ 18(0.5)	N	Y	NA	N	N	Surgical resection (Sx Rx)
15	92 M	IR AAA	↑AAA sac, back pain	TA	AAA sac at L4 level	34(2) + 18(1)	Coils	Y	Y	N	N	190
16	68F	IR AAA	↑AAA sac	TA	AAA sac	34(7) + 18(1)	Coils	Y	NA	N	N	Loss to follow up (LTF)
17	68F	IR AAA	↑AAA sac	TA	Lumbar A.	18(1)	N	Y	NA	N	N	LTF
18	73F	IR AAA	↑AAA sac	TA	AAA sac	34(9) + 18(1.5)	Coils	Y	Y	N	N	411

**Table 6:** Etiology: Arteriovenous malformations

No.	Age/Sex	Location of AVM	Presenting Sx/sign	Artery or target embolized	EVOH Conc (ml)	Adjunct embolic	TS	CS	NTE	Cx	FU (Days)
1	22F	Uterus	↑ vaginal bleeding	Right uterine A.	18(0.3)	N	Y	Y	N	N	90
2	40F	Uterus (Fig. 2)	↑ vaginal bleeding	Right & left uterine A.	18(0.9)	N	Y	Y	N	N	90
3	35F	Uterus	↑ vaginal bleeding	Right uterine A.	18(0.1)	N	Y	Y	N	N	30
4	48F	Left pelvic cavity	Pelvic pain	Two branches of ant division of left internal iliac artery (IIA)& one branch of uterine artery	34(0.6)	N	Y	N	N	N	90
5	49F	Left pelvic cavity	Pelvic pain	Third order branch of left external pudendal A. and a branch of anterior division of left IIA	34(1)	N	Y	Y	N	N	75
6	49F	Left pelvic cavity	Pelvic pain	Four distal branches of left IIA.	34(1.5)	Coil	Y	NA	N	N	LTF
7	50F	Left pelvic cavity	Pelvic pain	Branches of anterior division of left IIA	34(1)	Coil	Y	NA	N	N	LTF
8	40F	Left upper extremity	Pain/mass	Distal branches of ulnar A.	34(1.5)	N	Y	NA	N	N	Sx Rx
9	61 M	Right lower extremity	Calf pain	Peroneal A. branches	34(0.6)	N	Y	Y	N	N	439
10	61 M	Right lower extremity	Calf pain	Proximal PT branches	18(0.5)	N	Y	N	N	N	35
11	35 F	Calf; Congenital	Chronic calf pain	Muscular branch of peroneal A.	34(0.3)	N	Y	Y	N	N	77
12	35 F	Calf; Congenital	Chronic calf pain	AVM	18(1.3)	Sotradecol foam	Y	Y	N	N	70
13	35 F	Calf; Congenital	Chronic calf pain	AVM	18(3)	Sotradecol foam	Y	Y	N	N	79
14	72F	R thigh/ buttock	Pain, discomfort	Three branches of R profunda femoris A.	34(0.5)	N	Y	Y	N	N	90

**Table 7:** Etiology: Pseudoaneurys

No.	Age/Sex	Presenting Sx/sign	Artery or target embolized	Onyx Conc (ml)	Adjunct embolic	TS	CS	NTE	Cx	FU CT Days
1	61 M	Abdominal pain	Inferior pancreatico-duodenal A.	18(1)	N	Y	Y	N	N	30
2	28F	Abdominal pain (Fig. 3)	Left hepatic A. branch	18(0.6)	N	Y	Y	N	N	30
3	34 M	Abdominal pain	R hepatic A.	34(0.5)	Coils	Y	Y	N	N	37
4	53 M	Hematuria s/p L partial nephrectomy (Fig. 4)	Third order branch of left renal A.	34(1)	N	Y	Y	N	N	31
5	38F	R pelvic pain s/p total abdominal hysterectomy	Distal right uterine A.	34(0.8)	Coils	Y	Y	N	N	190
6	29 M	Asymptomatic, US surveillance s/p renal Bx	Right renal A.	34(0.5)	Coils	Y	Y	N	N	181
7	74 M	Hemodynamically unstable s/p L radical nephrectomy	Left renal A.	34(0.3)	N	Y	Y	N	N	248
8	73 M	Abdominal pain s/p right renal tumor resection	Two third order branches of inferior division of right renal A.	34(0.6)	N	Y	Y	N	N	235
9	29 M	Numbness & loss of dorsiflexion s/p stab injury to anterior calf	Proximal anterior tibial A.	34(0.4)	Coils	Y	Y	N	N	30
10	22 M	Right side hemothorax and pneumothorax after stab injury	Right upper lobe pulmonary A.	34(3)	Coils	Y	Y	N	N	30
11	63 M	Active intraperitoneal bleeding	Superior mesenteric A.	18(6)	N	Y	Y	N	N	31

**Table 8:** All remaining etiologies

No.	Age/Sex	Location	Presenting Sx/sign	Artery or target embolized	Onyx Conc (ml)	Adjunct embolic	TS	CS	NTE	Cx	FU Days
**Peripheral arteriovenous fistula (AVF)**											
1	28 M	Pelvic AVF after gun shot injury s/p right IIA ligation & gelfoam embolization of post division of left IIA.	Abdominal bleeding	Middle sacral A.	18(1)	Coils & gelfoam	Y	Y	N	N	42
2	80F	Right lower extremity (RLE)	RLE tissue loss	Three branches of profunda femoris	34(1.5)	N	Y	N	N	N	56
**Venous malformations (VM)**											
1	22 M	Chest wall	Pain and discoloration of right chest wall	VM	34 (1.3)	NBCA	Y	Y	N	N	105
2	22 M	Chest wall	Pain and discoloration of right chest wall	VM	18 (3)	NBCA	Y	Y	N	N	30
3	61 M	Right shoulder, back, pectoralis (Fig. 5)	Swelling of right shoulder, axilla & back	VM	34(1.5) + 18(4.5)	NBCA	Y	Y	N	N	Sx Rx
4	29 M	Plantar 3rd/4th intermetatarsal region	Left plantar foot pain	VM	18(1)	NBCA	Y	Y	N	N	Sx Rx
**Iatrogenic arterial rupture**											
1	52 M	Intra-abdominal	Metastatic colorectal carcinoma involving liver, Y-90 planning	Right Gastric A.	34(0.1)	N	Y	Y	N	N	37
2	63 M	Intra-abdominal	Metastatic colorectal carcinoma involving liver, Y-90 planning	Right Gastric A.	34(0.5)	N	Y	Y	N	N	298
**Malignancy**											
1	54 M	Right arm sarcoma (Fig. 6)	Right arm mass	Branches of brachial A. and profunda radialis A.	34(6)	Embospheres	Y	Y	N	N	Sx Rx
**Peripheral aneurysm**											
1	83 M	Popliteal A.	Enlarging left popliteal A. aneurysm sac	Left popliteal A. aneurysm sac	34(3)	N	Y	Y	N	N	227
**Acute gastrointestinal hemorrhage**											
1	57 M	Duodenal bulb ulcer (Fig. 7)	Hematemesis & malena	Proximal gastro-duodenal A.	34(0.5)	N	Y	Y	N	N	30

## Discussion

This retrospective review showed that EVOH is a safe embolic agent and has a valuable role in the treatment of various peripheral vascular pathologies including, but not limited to type II endoleaks, AVMs, VMs, postoperative peripheral PSAs, and arteriovenous (AV) fistulas.

Traditionally, administration of EVOH is considered technically complex and difficult to control. A steep learning curve of the embolization technique can result in an incomplete or nontarget embolization. This has likely limited the adoption of this embolic agent in the peripheral applications. Over a short period of use of EVOH at the current institution, high TS rates of EVOH embolization were achieved. The experience also suggested that EVOH can be safely used in the periphery.

Well-known within the literature, the delivery of Onyx involves a uniquely meticulous process (Guimaraes & Wooster, 2011; Kilani, 2015). Unlike *n*-butyl cyanoacrylate glue (NBCA), this nonadhesive agent does not attach to endothelium or microcatheters and has been shown to induce a very mild regional inflammatory reaction (Murayama et al., 1998; Duffner et al., 2002). Upon contact with the blood, slow solidification of EVOH in a centripetal fashion endows it with a foam like consistency. These properties allow slow, progressive and controlled deployment of the EVOH at the desired location leading to complete occlusion of desired targets of various sizes and shapes. Being a non-absorbable embolic agent, an irreversible target occlusion is achieved for permanent treatment of peripheral pathologies. However, permanent occlusion also raises the fear of healthy tissue necrosis in the event of nontarget embolization. As per authors’ experience, methodical techniques, adequate training and appropriate choice of EVOH viscosity play an important role for safe embolization and avoidance of complications. The EVOH with higher viscosity (Onyx 34) was chosen when the microcatheter tip was near the target and controlled injection was needed to prevent distal embolization. The lower viscosity EVOH (Onyx 18) was used when the target remained distant from the microcatheter tip or reaching the target proved challenging secondary to vessel tortuosity or increased vessel length. Lower viscosities of EVOH are safe for embolization of end arteries (Guimaraes & Wooster, 2011; Kilani, 2015). The size and flow rate of the target vessel was also considered in choosing the EVOH concentration.

Early literature reported higher rates (80%) of recanalization when only afferent arteries of the type 2 endoleaks (EL-2) were embolized with coils, without embolization of aneurysmal sacs, using transarterial (TA) approach. This retrospective study reported statistically significant lower rates (8%) of recanalization when aneurysmal sacs were embolized with coils using the translumbar approach (Baum et al., 2002). Recanalization through the interstices of coils could explain the incidence of recanalization with either approach. Another large (*n* = 84) retrospective study reported no statistically significant difference in the clinical success (i.e. absence of endoleak or aneurysm enlargement) rates of TA or TL approaches. In this study the TA approach included embolization of entire aneurysmal sac along with its afferent artery. The clinical success rates were 78% (18/23) when aneurysmal sac and afferent arteries were embolized with coils using TA approach, and 72% (45/62) when aneurysmal sac was embolized with coils/ NBCA using TL approach. (Stavropoulos et al., 2009) The study reported a complication i.e. nontarget embolization which could be related to the need for a rapid injection of NBCA to prevent its adhesion to the vessel or microcatheter tip. Another recent retrospective study showed no significant difference in aneurysm sac growth, persistent EL-2 or complications between TA and TL approaches. The study also reported technical success rate in 89% cases and absence of endoleak recurrence in 70% cases. The embolic agents used were NBCA, NBCA plus coils, or coils only (Yang et al., 2016). Few other studies reported good technical and clinical outcomes when EL-2 were embolized with EVOH or EVOH with other agents (Massis et al., 2012; Khaja et al., 2013; Marcelin et al., 2017; Abularrage et al., 2012). As per the current institutional experience, EVOH alone was sufficient to effectively occlude the afferent vessels as well as aneurysmal sac in 14/18 i.e. 78% of EL-2 cases. The 12 of total 18 EL-2 cases had prior endovascular interventions. Out of these twelve persistent EL-2 cases, EVOH alone was suffiecient in nine (75%) cases. In the remaining cases of new EL-2 (*n* = 1) and persistant EL-2 (*n* = 3), coils were used along with EVOH to occlude the aneurysmal sac. The approach for the embolization of EL-2 were either TA (89%) and TL (11%) with standard procedural steps as described before. (Bryce et al., 2018) The immediate post-embolization TS rate and CS rates were 100%. These results continue to support the use of EVOH in treatment of persistent EL-2 which are refractory to other embolic agents. The high TS rates are due to an ability of EVOH to penetrate and occlude afferent/efferent vessels and aneurysmal sacs of various sizes and shapes. Also the results such as absence of nontarget embolization or complications were similar to the results previously reported (Khaja et al., 2013; Marcelin et al., 2017; Abularrage et al., 2012).

For the treatment of new EL-2, the TA approach was preferred when access and embolization of aneurysmal sac via afferent vessels were possible. Once in the aneurysmal sac, whenever required based on the sac size, we first used coils to fill the aneurysmal sacs. Then EVOH was injected under fluoroscopic guidance until the proximal portions of efferent vessels were completely occluded. Then proximal portions of afferent vessels were occluded with EVOH while slowly withdrawing the microcatheter. When accessing an aneurysmal sac via afferent vessel using TA approach was not possible, the TL approach was used to directly enter the aneurysmal sac and EVOH was injected until aneurysmal sac and proximal portions of afferent and efferent vessel were completely occluded. In the cases of recurrent EL-2 (*n* = 12), the aneurysmal sacs were already partially occluded with prior embolic material, hence in most of these cases (75%) EVOH alone was sufficient to achieve complete occlusion. The preference of approach and technique of embolization were similar to the management of a new EL-2 as described before. As per this experience, EVOH was an ideal embolic agent when the target cannot be directly catheterized, for example if the route was tortuous, small in caliber or distal to negotiate with a microcatheter. In complex cases of EL-2 when access to culprit afferent vessel by TA approach or access to aneurysmal sac via TL approach were not possible, then low viscosity EVOH (Onyx 18) was injected in the proximal aspects of afferent vessels. Other endovascular approaches such as transcaval (Giles et al., 2015) and perigraft (Coppi et al., 2014), though not used in our study, can be useful.

Uterine AVMs are rare but potentially life threatening. Similar to a prior case series, this retrospective review showed 100% TS and CS rates of EVOH embolization of three high flow AVMs in women of reproductive age group (22 to 40 years). At the current institution, the use of low viscosity EVOH (Onyx 18) allowed selective embolization of the niduses of uterine AVMs that were difficult to reach with the microcatheters. None of the patients underwent subsequent hysterectomy. There is no data reporting the most appropriate embolic agent to treat uterine AVMs. The available data indicates that resorbable agents and coils are ineffective. (Barral et al., 2017)

EVOH is considered ideal for embolization of AVMs due to it's ability to penetrate and conform to the shapes of tortuous afferent arteries and variable nidus sizes. Being a non-adhesive agent, it allows precise positioning and control of the tip of the delivery microcatheters during the EVOH injections. The Interventionalist can interrupt the injection, reanalyze the EVOH cast and reinject at the new location to occlude a large AVM without filling the draining veins (Regine et al., 2015). A prior study has reported reflux of EVOH within the afferent artery during embolization of high flow AVM and nontarget embolization. This was attributed to complex angio-architecture, short arterial feeders close to parent arteries and poor radiopacity of earlier generation of EVOH. During the final stages of EVOH injection in the afferent arteries, the pressure reaches critical threshold and EVOH can reflux into the afferent artery. However with continuous fluoroscopic monitoring, interventionalists can modulate the volume and rate of injection to effectively occlude the afferent artery and avoid the reflux. Also, the use of higher viscosity EVOH to embolize the afferent arteries of high flow AVMs can cause an instant onsite polymerization and occlusion. This can avoid the further passage of EVOH through fistulous component and venous reflux (Cantasdemir et al., 2012).

In this series, the complex or large venous malformations (VM) were percutaneously embolized with a combination of liquid embolic agents; NBCA for the superficial component and EVOH for the deeper component of VM (Fig. 2). As described in the recent literature (Salaskar et al., 2020), the use of EVOH is advantageous in the embolization of deeper parts of complex VM for several reasons. Being a nonadhesive agent, EVOH could be precisely delivered in the deeper aspects of VMs. After intravascular precipitation, the deeper aspect of VM retained a soft sponge-like consistency. This facilitated surgical handling during resection of VM. The use of EVOH embolization has been previously shown to be superior for surgical resection of AVMs when compared with NBCA embolization (Akin et al., 2003). EVOH was shown to incur minimal intra or perivascular inflammatory reaction (Murayama et al., 1998; Duffner et al., 2002). Similarly absence of inflammation facilitated safe surgical resection of complex VMs. To prevent non target embolization, high viscosity EVOH was used when pre-embolization venography of VM revealed contrast entry into the central veins. The conventional alcohol sclerotherapy of VM has side effects such as tissue swelling, mucosal blistering, necrosis, and neuropathy (Cantasdemir et al., 2012). None of these side effects were observed with the use of EVOH.

Few case reports have described the use of EVOH in the treatment of renal PSAs and AV fistulas (Vanninen & Manninen, 2007; Carberry et al., 2013; Zeleňák et al., 2009). In this series, four cases of post-surgical renal PSAs were successfully embolized, including an urgent case in which successful cessation of life threatening bleeding was achieved by EVOH embolization of a post nephrectomy PSA (Fig. 4). In these cases, EVOH embolization involved filling the afferent & efferent arteries as well as the PSA sac. In order to prevent distal nontarget embolization, high viscosity EVOH was used.

EVOH embolization has also been used in the treatment of active gastrointestinal hemorrhage with good outcomes (Kolber et al., 2015; Lenhart et al., 2010). The results of one prior retrospective series demonstrated 100% TS rate without any complications in patients who underwent EVOH embolization for the treatment of persistent gastrointestinal bleeding despite endoscopic interventions (Lenhart et al., 2010). Unlike other embolic agents such as coils, EVOH polymerization does not depend on a functional coagulation cascade. This property is pivotal in controlling the active bleeding in patients with underlying coagulopathies (Müller-Wille et al., 2011; Carberry et al., 2013). In the management of iatrogenic & traumatic arterial ruptures, EVOH was preferred to occlude the damaged target vessel which could not be catheterized directly. Also the ability to deploy EVOH without exerting any radial pressure to the damaged vessel walls makes EVOH an ideal agent.

Previously reported DMSO related side effects such as local pain after rapid injection of Onyx (Vanninen & Manninen, 2007), foul breath, severe respiratory distress, pulmonary edema due to DMSO related oxygen desaturation (Pamuk et al., 2005; Murugesan et al., 2008), cardiovascular instability secondary to vasovagal reaction from irritation of nociceptive nerve fibers of intercostal arteries and/or aortic side branches by DMSO (Wildgruber et al., 2016) were not seen in this study group. As per the current experience, use of an appropriate amount of DMSO to adequately fill the lumen of the microcatheter can avoid these risks. The tantalum powder in the mixture of Onyx may cause streak artifacts on radiographic and CT images. This may hinder visualization of future recurrence or regional tissues (Jia et al., 2015). On MR images, EVOH appears hypointense and does not cause artifacts.

Prior study has reported longer fluoroscopy and procedure time with EVOH embolization when compared to those with NBCA embolization. This was attributed to the slow injection rate of EVOH, however in our experience these are operator dependent. The slow, controlled injection of EVOH is in fact desirable for precise and effective embolization (Velat et al., 2008).

The perceived high cost of EVOH embolization may limit its adoption. A vial of EVOH costs approximately $2000 USD. A vial of NBCA is approximately the same cost at the institute. The average cost of total EVOH used per case is estimated to be approximately $4000 USD at our institute. The use of EVOH can sometimes leads to reduction in the use of coils, thereby leads to cost savings. However, if multiple vials of EVOH or coils are required for the procedure, the cost advantage can be quickly lost. Therefore each case should be prudently planned to preserve resources.

The limitations of this study are its retrospective nature and small sample size. Ideally the safety and efficacy of EVOH embolization should continue to be evaluated by comparing it to standard embolic therapies in prospective studies.

## Conclusion

This single institutional experience supports the safety and efficacy of EVOH embolization in the treatment of various peripheral vascular pathologies.

## Data Availability

Not applicable.

## Abbreviations

EVOH: Ethylene vinyl alcohol
AVF: Arteriovenous malformation
PSA: Pseudoaneurysms
VM: Venous malformations
TS: Technical success
CS: Clinical success
NTE: Non target embolization
